# Guided antipsychotic reduction to reach minimum effective dose (GARMED) in patients with remitted psychosis: a 2-year randomized controlled trial with a naturalistic cohort

**DOI:** 10.1017/S0033291723000429

**Published:** 2023-11

**Authors:** Chen-Chung Liu, Ming H. Hsieh, Yi-Ling Chien, Chih-Min Liu, Yi-Ting Lin, Tzung-Jeng Hwang, Hai-Gwo Hwu

**Affiliations:** 1 Department of Psychiatry, National Taiwan University Hospital, Taipei, 10002, Taiwan; 2 Department of Psychiatry, College of Medicine, National Taiwan University, Taipei, Taiwan

**Keywords:** Antipsychotics, functioning, minimum effective dose, remission, tapering

## Abstract

**Background:**

Patients with remitted psychosis face a dilemma between the wish to discontinue antipsychotics and the risk of relapse. We test if an operationalized guided-dose-reduction algorithm can help reach a lower effective dose without increased risks of relapse.

**Methods:**

A 2-year open-label randomized prospective comparative cohort trial from Aug 2017 to Sep 2022. Patients with a history of schizophrenia-related psychotic disorders under stable medications and symptoms were eligible, randomized 2:1 into guided dose reduction group (GDR) *v.* maintenance treatment group (MT1), together with a group of naturalistic maintenance controls (MT2). We observed if the relapse rates would be different between 3 groups, to what extent the dose could be reduced, and if GDR patients could have improved functioning and quality of life.

**Results:**

A total of 96 patients, comprised 51, 24, and 21 patients in GDR, MT1, and MT2 groups, respectively. During follow-up, 14 patients (14.6%) relapsed, including 6, 4, and 4 from GDR, MT1, and MT2, statistically no difference between groups. In total, 74.5% of GDR patients could stay well under a lower dose, including 18 patients (35.3%) conducting 4 consecutive dose-tapering and staying well after reducing 58.5% of their baseline dose. The GDR group exhibited improved clinical outcomes and endorsed better quality of life.

**Conclusions:**

GDR is a feasible approach as the majority of patients had a chance to taper antipsychotics to certain extents. Still, 25.5% of GDR patients could not successfully decrease any dose, including 11.8% experienced relapse, a risk comparable to their maintenance counterparts.

## Introduction

Patients with psychotic disorders wish to have a chance to withdraw from antipsychotics entirely after achieving symptom remission (Perkins et al., 2008). Not only patients but also a proportion of clinicians thought it practicable to discontinue antipsychotics once remission has been achieved (Hui et al., 2019; Thompson, Singh, & Birchwood, 2016; Yen et al., 2022). Nowadays, more patients can achieve remission by early intervention after first-episode psychosis (Lally et al., 2017), but the much higher risks of relapse following medication discontinuation do not endorse discontinuing antipsychotics when their symptoms have remitted (Correll, Rubio, & Kane, 2018; Robinson et al., 1999). Even though there is evidence advocating that a minority of patients could remain drug-free and do well in the long term (Harrow, Jobe, & Faull, 2012; Wils et al., 2017; Wunderink, Nieboer, Wiersma, Sytema, & Nienhuis, 2013), and the potential hazards of long-term antipsychotic treatment to patient's brain structure, cognitive functioning, and physical health can never be overlooked (Fusar-Poli et al., 2013; Ho, Andreasen, Ziebell, Pierson, & Magnotta, 2011; Vita, De Peri, Deste, Barlati, & Sacchetti, 2015; Voineskos et al., 2020), the difficulty in finding valid predictors to identify who can successfully discontinue antipsychotics (Alvarez-Jimenez et al., 2012; Bowtell, Ratheesh, McGorry, Killackey, & O'Donoghue, 2018b) keeps the argument between maintenance *v.* discontinuation of antipsychotics for patients with remitted psychosis in controversies.

While McGorry, Alvarez-Jimenez, and Killackey (2013) have postulated that ‘less (*antipsychotic*) might be more (*benefiting*)’ to patients with remitted psychosis, theoretically, an ideal approach is to treat patients with ‘the lowest effective dose’ to optimize the risk-to-benefit ratio of antipsychotic treatment. However, there was no pragmatic guide available for ensuring safe dose tapering until Wunderink *et al*.'s (2013) puzzling findings, which implied that even though patients undergoing dose reduction had a higher initial rate of relapse, their functioning was better after 7 years of follow-up compared to their antipsychotic maintenance counterparts. Since then, several clinical trials were initiated to test if it is feasible to conduct dose reduction to reach medication discontinuation, and eventually to achieve better outcome (Begemann et al., 2020; Moncrieff et al., 2019; Sturup et al., 2017; Weller et al., 2019). While all these trials anticipated the discontinuation group to regain better functioning, they also recognized the risk of having higher relapse rates during the process of medication discontinuation compared to the dose maintenance group.

To mitigate such an undesirable tradeoff, we proposed an alternative approach beyond the dichotomy between maintenance and discontinuation of antipsychotics with a subtle yet pivotal fine-tuning of the attitude toward the role of antipsychotics in patients with remitted psychosis. That is, not aiming at complete medication discontinuation but anticipating a chance to reduce their antipsychotics to the lowest effective dose (Liu & Takeuchi, 2020).

Previously, the minimum effective dose (MED) was estimated by the dose which could exhibit a differentiable effect of reducing symptom severities in randomized, double-blind, fixed-dose trials comparing antipsychotics to placebo (Leucht, Samara, Heres, & Davis, 2016; Woods, 2003); however, MED in stable patients has never been well explored. Our naturalistic longitudinal observation study revealed that a substantial proportion of patients could achieve good functioning under low-dose antipsychotic [⩽ 200 mg/d chlorpromazine equivalent (CPZE) dose] maintenance after first-episode psychosis, even if they could not completely discontinue antipsychotics (Liu et al., 2021). Indeed, a series of studies have suggested that the therapeutic window of D2 receptor blockade could be lower than 65% for stable patients with schizophrenia (Graff-Guerrero et al., 2015; Tsuboi et al., 2015; Uchida et al., 2014), implying that the maintenance dose could be lower than the previously recommended MED levels.

On the other hand, it would be unwise to reduce dose inadvertently, as dopamine supersensitivity-mediated relapse (Chouinard et al., 2017) and certain antipsychotic-withdrawal syndromes (Horowitz, Murray, & Taylor, 2022b) might mislead to the judgment of higher relapse rates in clinical trials in which antipsychotics were discontinued within a few weeks. Thus, instead of looking at completely discontinuing medications, we proposed a procedure to reduce a small fraction of the current dose at a time recursively, which was inspired by the metaphor of the Cantor set from natural philosophy [see online Supplement Fig. S1a and the rationale detailed in an article published in CNS Drugs 2020 (Liu & Takeuchi, 2020)]. This procedure can be run indefinitely by empowering patients with shared decision-making to determine a suitable time and tempo of the next dose reduction, consequently the risk of relapse can be minimized. And via this procedure, someday some patients can taper down their antipsychotic dosage to as low as ‘approximate to zero’ as illustrated by the Cantor's set, which will be a mathematical equivalent to medication discontinuation.

Derived from such a notion, a protocol of guided antipsychotic reduction to reach minimum effective dose (GARMED) based on a pragmatic design was operationalized and employed in real world setting at the study hospital from 2017 (Liu et al., 2022). In this report, we focused on (1) if any difference in the rates of relapse between guided dose reduction (GDR) and maintenance treatment (MT) groups, (2) the chance of staying remitted under a dose lower than baseline levels and to what extent the dose could be reduced in GDR group, and (3) if GDR patients could exhibit improved psychosocial functioning and quality of life, by the end of 2-year follow-up.

## Methods

### Design, setting, and participants

This is an open-label randomized prospective comparative cohort trial based on a pragmatic design (online Supplement Fig. S2) (Relton, Torgerson, O'Cathain, & Nicholl, 2010). This manuscript is written in line with the Consolidated Standards of Reporting Trials statement (Schulz, Altman, & Moher, 2010).

Patients with remitted psychosis receiving regular treatment at the outpatient or daycare services of a university-affiliated teaching hospital were invited to participate in this study from August 2017 to September 2022. Patients eligible for this study yet declined to participate in the dose reduction trial were invited to receive prospective follow-up under treatment as usual. Ethical approval has been obtained from the Research Ethics Committee of the study hospital, (REC: 201703002RIND) and this study was registered at ClinicalTrials.gov (identifier: NCT03248180).

### Eligibility of participants

Stable male and female outpatients or patients at psychiatric daycare service, age 18–60 years old, with a diagnosis of schizophrenia, schizophreniform disorder, or other schizophrenia-spectrum and other psychotic disorder based on the DSM-5 criteria, currently receiving antipsychotic treatment at a fixed dose for at least 3 months (including long-acting injectable antipsychotic), currently having PANSS positive and general symptom score ⩽ 3 in P1: delusion, P2: conceptual disorganization, P3: hallucination, G9: unusual thought, G5: mannerism and posturing, and negative symptom score ⩽ 4 in N1: blunted affect, N4: social withdrawal, N6: lack of spontaneity/flow in conversation for at least 3 months, with no revised use of benzodiazepines, antidepressants, anticholinergics, or other concomitant medications during the past 3 months and a second antipsychotic agent only used for a low-dose, as needed adjuvant purpose were eligible to participate in this trial.

Patients with a score ⩾ 5 on any of the 30 PANSS rating items at screening, admission to the acute psychiatric unit during the past 6 months, a change in dose of current antipsychotic medication in recent 3 months, concomitant use of mood stabilizers, such as lithium, valproic acid, or other anti-epileptic drugs, IQ below 70 prior to the diagnosis of schizophrenia, a history of pervasive mental disorder or bipolar disorder, a medical condition with significant cognitive sequelae, a history of substance dependence during the past 6 months, or currently in pregnancy or breastfeeding were not eligible to this trial.

Eligible patients were briefed with a psychoeducation session regarding the possible risk and benefit of long-term antipsychotic treatment and the rationale of this GDR trial before obtaining their written informed consent. Patients younger than the age of 20 years old needed to provide written consent from their parents in accordance to Taiwan's law.

### Randomization and subgrouping

**Fig. 1.**
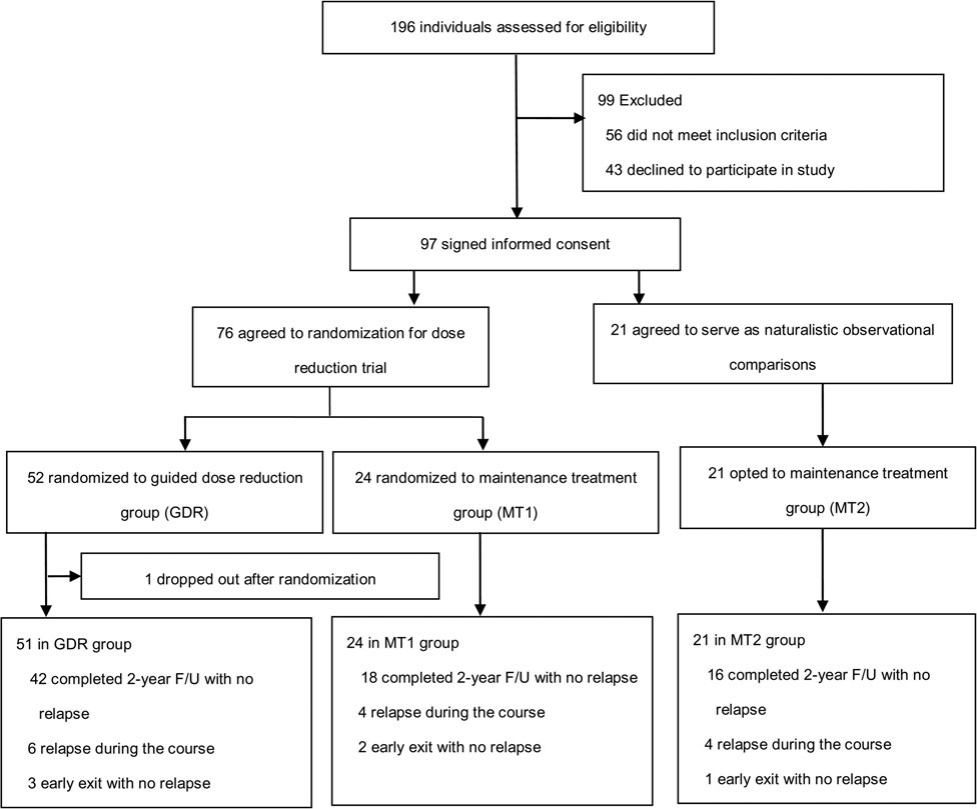
Diagram of trial flow chart.

Eligible patients to try dose reduction were randomized with a 2:1 ratio into a GDR group *v.* maintenance treatment group (MT1), together with a group of eligible patients who volunteered to stay at maintenance treatment, serving as a naturalistic comparison group (MT2) which respecting patient preferences in recruitment and allowing better generalizability of outcomes to real world practice (Relton et al., 2010). The flow chart of study design is illustrated in Fig. 1.

### Dose-reduction algorithm

For GDR patients, more instructions were delivered regarding the extent and the tempo of dose reduction, warning signal of relapse, timing to call for help if in need to resume rescue dose, and a shared decision-making process during tapering (summarized in online Supplement Table S1).

In the beginning, no more than 25% of the baseline antipsychotic dose was reduced at a time. Patients needed to be monitored every 4 weeks (or 1 month) for 3 consecutive sessions once undergoing dose reduction. They would stay at the same dose for another 12 weeks (or 3 months) if maintained stabilized. Thus, the next dose reduction attempt would not be initiated until 24 weeks (or 6 months) of stabilization, defined as no aggravation of symptoms lasting for more than one week during this span. When the patient was eligible to consider next dose reduction, they were empowered to take shared decision-making as they might opt to stay at their current dose for a more extended time if not feeling ready for continuing dose reduction for any reason.

The subsequent dose reduction was a reiteration of the previous step, cutting off one-quarter of the current dose, yielding 9/16 (3/4 × 3/4) of baseline dose, rather than cutting off another 1/4 of the baseline dose, as the latter would result in reducing 1/2 (1/4 + 1/4) of the baseline dose after 2 dose-reductions. Importantly, the second formula will *end to zero* by 4 dose reducing steps, while the first formula ((3/4)^n^) can be *re-iterated indefinitely*. Thus the dose was reduced in a hyperbolic rather than a linear manner, mathematically equivalent to the Cantor's formula for Sierpinski triangle (online Supplement Fig. S1b), a strategy suggested to be a better approach based on findings of association between antipsychotic dose and D2 receptor occupancy by PET studies, to minimize negative impact of dopamine-supersensitivity and related withdrawal symptoms (Horowitz, Jauhar, Natesan, Murray, & Taylor, 2021a; Horowitz, Murray, & Taylor, 2021b). The actual dose taken in our GDR patients would not always be precisely the number calculated by the algorithm as it was impractical to cut off a quarter or even smaller piece of a tablet for daily dosing. Simulating intermittent dosing strategies in previous studies (Remington et al., 2011), we generated several versions of intermittent or irregular dosing schedules to meet the needs based on pragmatic considerations (online Supplement Table S2) (Liu et al., 2022).

### Measurements of major outcomes

All patients were required to keep a log of their actual medication administration daily due to possible errors while undergoing an irregular dosing schedule. Patients could contact the study team during the course whenever they felt unsure if any relapse sign might be re-emerging, such as anxiety, irritability, restlessness, oversensitive to interpersonal or perceptual cues, and sleep disturbance (Yang, Shih, Yeh, & Chen, 1996). The study team would arrange extra visits for evaluation and supervise GDR patients to resume dosage at the previous level once an impending relapse was suspected, and then closely monitored if symptoms could be stabilized within one week. If a patient's symptoms could not be stabilized within one week (that is, any one of the 3 positive symptoms and 2 general symptoms in the inclusion criteria scores > 3) under an antipsychotic dose equal to their baseline dose, the patient was designated as having a relapse and would be treated with a dose higher than their baseline level. The percentage of dose reduced at a designated time point was calculated by the formula [1- (current dose)/(baseline dose)] × 100%.

### Other assessments

Clinical assessments, including clinical severities rated by the Mandarin version Positive and Negative Syndrome Scale (PANSS) (Cheng, Ho, Chang, Lane, & Hwu, 1996), Clinical Global Impression of Severity (CGI-S), and Personal and Social Performance scale (PSP) (Wu et al., 2013) were done every 4 weeks for 3 times after a dose reduction attempt by the patient's attending psychiatrist; otherwise rated every 12 weeks or if any early return for signs of suspected relapse. Patients reported subjective experiences by filling a medication satisfaction questionnaire (MSQ) comprised a 7-point Likert scale (Vernon et al., 2010) and checking on the EuroQoL-5D visual analog scale (EQ-5D-VAS) for quality of life (Chang et al., 2013).

Physical examinations, including body weight, body height, waist girth, and blood pressure were measured at baseline and the end of the study. Besides, we reviewed the patient's medical records to collect demographics, clinical diagnosis, age of onset, illness duration, and any history of psychiatric hospitalization and psychotic relapse during their illness.

### Statistical considerations

The baseline clinical characteristics among groups were compared using χ^2^ tests for the categorical variables and analyses of variance to examine differences in continuous variables. A summary of dose reduction was displayed by descriptive statistics. The survival function during follow-up was examined by Kaplan-Meier survival estimates. The relative risk of time-to-relapse between groups was examined by Cox Proportional Hazards (PH) with age, gender, duration of illness, diagnosis of schizophrenia or other psychotic disorder, history of admission, history of relapse, baselines PANSS score and CPZE dose as covariates.

All longitudinal outcome analyses were performed based on the intent-to-treat population with missing data being imputed using the last observation carried forward approach. The changes in PANSS, CGI, PSP, and EQ-5D-VAS scores were analyzed by paired *t* test comparing the difference in scores between baseline and 2-year follow-up. Hypothesis testing and confidence intervals (CIs) were two-sided at the 5% significance level. If the relapse rates of GDR and MT1 were 20% and 10%, respectively, set the type I error at 5% and the power at 0.8, the estimated sample sizes were 47(GDR) and 23(MT1) for one-sided comparison. (STATA 13; Stata Corp., College Station, Texas 77845 USA).

## Results

### Baseline characteristics

A total of 97 patients agreed to participate in this study. Excluding 1 patient who did not show up after randomization, 96 patients underwent this 2-year trial, including 51, 24, and 21 patients from GDR, MT1, and MT2 groups; among them, only 3, 2, and 1 patient from each group, respectively, exited prematurely not due to relapse (Fig. 1). Statistically no significant differences in patient's baseline demographic and clinical characteristics, although the GDR group seemed to be younger and with shorter duration of illness (Table 1). Patient's stable remitted states were shown by their low baseline symptoms and global severity scores, and relatively high personal social functioning. The illness duration ranged widely from 0.8 to 38 years, an average of 11.3 ± 8.4 years. The majority of them had a diagnosis of schizophrenia or schizophreniform disorder (78.1%), a history of psychiatric hospitalization (56.3%) and a history of relapse (67.7%) during their illness. Their baseline treatment comprised 12 oral and 3 long-acting injectable antipsychotics with dose ranges skewed to the lower end of each agent with an average of CPZE dose around 200 mg/d (online Supplement Table S3).

**Table 1.** Baseline demographic and clinical characteristics of participants

	GDR (*n* = 51)	MT1 (*n* = 24)	MT2 (*n* = 21)	Total (*n* = 96)	*p* value
Age, Years old, (s.d.)	34.3 (9.6)	39.1 (11.0)	38.2 (10.3)	36.3 (10.2)	0.106
Gender, Male (%)	26 (51.0%)	7 (29.2%)	10 (47.6%)	43 (44.8%)	0.199
Employment ^a^					0.142
Full-time	18	14	12	44	
Part-time	19	4	3	26	
No	14	6	6	26	
BMI (s.d.)	26.0 (4.8)	24.8 (3.1)	25.3 (5.2)	25.6 (4.5)	0.494
Diagnosis					0.342
Schizophrenia/schizophreniform	41	20	14	75	
Other schizophrenia spectrum/other psychotic disorders	10	4	7	21	
Duration of illness, years (s.d.)	9.6 (8.5)	14.6 (8.6)	11.7 (7.3)	11.3 (8.4)	0.055
With a history of hospitalization (%)	29 (56.9%)	13 (54.2%)	12 (57.1%)	54 (56.3%)	0.972
With a history of relapse (%)	34 (66.7%)	18 (75%)	13 (61.9%)	65 (67.7%)	0.627
CPZE mg/d, (s.d.)	206.7 (134.1)	169.3 (119.2)	221.8 (128)	200.6 (129.3)	0.356
PANSS total score (s.d.)	40.5 (9.7)	40.4 (7.9)	42.7 (7.1)	41.0 (8.7)	0.605
CGI-S score (s.d.)	2.04 (0.80)	1.96 (0.91)	1.81 (0.6)	1.97 (0.79)	0.535
PSP score (s.d.)	80.1 (8.4)	80.2 (8.6)	81.9 (5.7)	80.5 (7.9)	0.664
EQ-5D-VAS (s.d.)	75.1 (12.8)	74.5 (15.8)	71.4 (10.7)	74.1 (13.2)	0.558
MSQ score (s.d.)	5.27 (1.27)	5.58 (1.10)	5.43 (0.75)	5.39 (1.13)	0.536

Abbreviations: BMI, body mass index; CGI-S, clinical global impression-severity; CPZE chlorpromazine equivalent dose; EQ-5D-VAS, EuroQoL-5D visual analogue scale; GDR, patients randomized to guided dose reduction group; MSQ, medication satisfaction questionnaire; MT1, patients randomized to maintenance group; MT2, patients volunteered to be the naturalistic observational group; PANSS, positive and negative syndrome scale; PSP, personal and social performance.

*p* value denotes the statistics among 3 groups.

a Full-time employment defined as having a job > 15 h per week for more than 3 months during the past 6 months; Part-time employment defined as serving some duties or having a job but not meeting the requirement for full-time employment.

### Relapse rates during follow-up

**Fig. 2.**
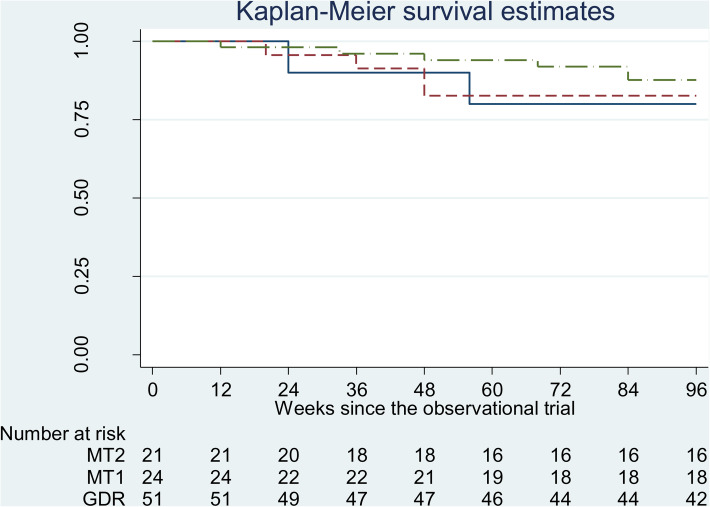
Kaplan-Meier survival estimates of proportions of patients who remained in remission during follow-up. The long dash-dot line represents the guided dose reduction group (GDR), the dashed line represents the Maintenance Group 1 (MT1), and the solid line represents the naturalistic observational comparison group (MT2).

A total of 14 patients (14.6%) relapsed during follow-up, including 6 (11.8%), 4 (16.7%), and 4 (19%) from GDR, MT1, and MT2 groups, respectively. Illustrated by the Kaplan-Meier survival curve and examined by Cox regression, Breslow method for ties, there is no significantly different risk of relapse between groups (LR χ^2^(2) = 0.83, Log likelihood = −61.93; *p* = 0.66) (Fig. 2). Comparing GDR to the other 2 maintenance groups separately, the hazard ratio (HR) of relapse between GDR and MT1 is 0.659 [95% CI 0.186~2.337, *p* = 0.526] and the HR between GDR and MT2 is 0.577 (95% CI 0.163~2.046, *p* = 0.406). The risks of relapse between GDR and MT1 as well as GDR and MT2 were not significantly different after taking into account baseline demographic and clinical variables.

In this cohort, no demographic or clinical variables were significantly related to the risk of relapse except baseline PANSS scores; as adjusted for gender and baseline CPZE, higher baseline PANSS scores were associated with increased risk of relapse (HR 1.06, 95% CI 1.01~1.11, *p* = 0.021).

### The chance and the extent of dose reduction

The trajectories and extent of actual dose tapering of the GDR group varied widely (view example illustrations in online Supplement Fig. S3). Among them, by the end of 2-year follow-up, 18 patients (35.3%) completed 4 consecutive dose tapering steps as designated by our proposed algorithm, on average reduced to 40% of their baseline dose; 20 patients (39.2%) took 1 to 3 steps of dose tapering during the course and were able to stay at reduced dose levels (50% to 80% of baseline); 7 patients (13.7%) eventually needed to resume their baseline dose in precaution as to prevent from full-blown relapse; 6 patients (11.8%) experienced relapse, including 2 of them being hospitalized.

In total, 74.5% of GDR patients maintained in remission with no relapse under a dose lower than their baseline levels, on average 39% of baseline dose reduced. For comparison, the MT1 patients who were interested in taking dose reduction but designated to the maintenance group also on average successfully reduced 11% of their baseline dose by the end of 2 years on their own accord; while the MT2 patients who preferred to take a conservative stance stayed with their baseline dose (Table 2).

**Table 2.** Comparison of differences in clinical scores at baseline and by the end of completing 2 years follow-up among 3 groups

	GDR (*n* = 42)	MT1 (*n* = 18)	MT2 (*n* = 16)	*p* value
PANSS total (s.e.), BL	38.83 (0.96)	39.89 (1.97)	42.94 (1.84)	
PANSS total (s.e.), 2Y	36.90 (0.81)	38.22 (1.96)	40.50 (1.51)	
Difference (95% CI)	−1.93 (−3.65 to −0.2)	−1.67 (−2.82 to −0.51)	−2.44 (−4.52 to −0.35)	0.886
*p* value by paired *t* test	0.029	0.007	0.025	
CGI-S score (s.e.), BL	1.98 (0.12)	1.78 (0.21)	1.81 (0.14)	
CGI-S score (s.e.), 2Y	1.74 (0.11)	1.78 (0.21)	1.75 (0.11)	
Difference (95% CI)	−0.24 (−0.40 to −0.07)	0	−0.06 (−0.2 to 0.07)	0.091
*p* value by paired *t* test	0.006	~	0.333	
PSP score (s.e.), BL	80.98 (1.09)	81.33 (2.16)	81.81 (1.35)	
PSP score (s.e.), 2Y	83.76 (0.94)	82.22 (2.12)	82.81 (1.15)	
Difference (95% CI)	2.78 (1.26 to 4.31)	0.89 (0.17 to 1.61)	1 (−0.66 to 2.66)	0.143
*p* value by paired *t* test	0.001	0.019	0.219	
EQ-5D-VAS (s.e.), BL	74.71 (2.00)	74.83 (3.80)	72.81 (2.33)	
EQ-5D-VAS (s.e.), 2Y	80.79 (2.13)	78.22 (3.32)	75.5 (3.62)	
Difference (95% CI)	6.07 (1.61 to 10.53)	3.39 (−3.06 to 9.83)	2.69 (−4.41 to 9.78)	0.634
*p* value by paired *t* test	0.009	0.283	0.432	
MSQ score (s.e.), BL	5.29 (0.20)	5.44 (0.26)	5.5 (0.2)	
MSQ score (s.e.), 2Y	5.55 (0.15)	5.61 (0.20)	5.69 (0.22)	
Difference (95% CI)	0.26 (−0.05 to 0.58)	0.16 (−0.18 to 0.52)	0.19 (−0.21 to 0.59)	0.917
*p* value by paired *t* test	0.102	0.331	0.333	
Mean dose for reference				
CPZE mg/d (s.d.), BL	208.9 (137.1)	180.7 (127.4)	228.8 (132.7)	
CPZE mg/d (s.d.), 2Y	123.3 (80.3)	160.7 (125.6)	228.8 (132.7)	

Abbreviations: 2Y, the end of 2-year follow-up; BL, baseline; CGI-S, clinical global impression-severity; CPZE chlorpromazine equivalent dose; EQ-5D-VAS, EuroQoL-5D visual analogue scale; GDR, patients randomized to guided dose reduction group; MSQ, medication satisfaction questionnaire; MT1, patients randomized to maintenance group; MT2, patients volunteered to be the naturalistic observational group; PANSS, positive and negative syndrome scale; PSP, personal and social performance.

### Impacts of dose reduction to clinical outcomes

Compared to their baseline scores by paired *t* test, the GDR patients showed a statistically significant decrease of PANSS score by 1.93 points [Standard Error (s.e.): 0.85] at 96 weeks (*p* = 0.029), which is parallel to the extent of decrease in symptom severity of the other 2 groups (MT1: −1.67, s.e. 0.55, *p* = 0.007; MT2: −2.44, s.e. 0.98, *p* = 0.025). GDR is the only group to show improved CGI score (from 1.98 to 1.74, s.e. 0.08, *p* < 0.01). Both GDR and MT1 had improved PSP scores (2.79, s.e. 0.76, *p* = <0.001 and 0.89, s.e. 0.34, *p* = 0.02, respectively). Subjectively, only the GDR patients reported significantly better quality of life, manifested by an increase of EQ-5D-VAS scores from 74.7 to 80.8 (s.e. 2.21, *p* = 0.009) (Table 2).

## Discussion

This is one of the few studies to test guided antipsychotic dose reduction in patients with remitted psychosis (Huhn et al., 2021; Wunderink et al., 2007). Based on a pragmatic study design with the inclusion of a naturalistic observational subgroup which provided treatment-as-usual comparisons (Relton et al., 2010), together with an operationalized algorithm implementable in real world setting, our results create a compromised solution beyond the dichotomy between antipsychotic maintenance and discontinuation while evaluating the risk-to-benefit ratio for treating patients with stable psychosis. Most importantly, our algorithm exemplifies how to taper antipsychotic treatment ‘very slowly and in a hyperbolic manner’ as suggested by Horowitz et al. (2021b), with a few highlights to emphasize.

First, we could successfully manage to keep the relapse rates among patients conducting dose reduction not higher than that of 2 maintenance groups, while the other 4 ongoing dose reduction/discontinuation trials anticipating higher relapse rates as the payoff of medication discontinuation (Begemann et al., 2020; Moncrieff et al., 2019; Sturup et al., 2017; Weller et al., 2019), not to mention previous clinical trials all showing significantly higher relapse rates in discontinuation groups (Chen et al., 2010; Emsley, Oosthuizen, Koen, Niehaus, & Martinez, 2012; Gaebel et al., 2011; Mayoral-van Son et al., 2016; Wunderink et al., 2007). In fact, in this cohort, 4 of the 14 patients (2 from GDR, 2 from MT1) with relapse confessed that they conducted dose reduction faster than our designated schedule. Thus, we believe that the seemingly modest goal of not aiming at complete withdrawal from antipsychotics and the conservative approach with a pre-requisite of 6-month stabilization to be pivotal for reaching this balanced outcome. Even though intermittent/irregular dosing strategy was inferior to the regular dosing schedule in previous literature (De Hert et al., 2015; Gaebel et al., 2011; Sampson, Mansour, Maayan, Soares-Weiser, & Adams, 2013; Shimomura et al., 2020), our results suggested that close monitoring and supervision during tapering could mitigate its pitfalls.

Second, a substantial proportion of GDR patients (39.2%) did not conduct successive dose reductions while being eligible for the next dose reduction for a variety of reasons. Since many of our patients had a history of relapse or hospitalization, they were likely to weigh the risk-to-benefit ratio more cautiously. This might be another key to keep the relapse rate of the GDR group not higher than the maintenance group. Respecting patient's autonomy, empowerment of patients and their principal caregivers to participate in shared decision-making could assure if they were ready for the next tapering attempt as to minimize the potential risk of relapse attributable to non-pharmacological factors, such as psychosocial stressors (Horowitz et al., 2022a). Without such an active involvement in treatment, even considering re-escalating dose to previous levels at the emergence of suspected early warning signs, the relapse rates or the proportion of patients needed to resume baseline doses might be higher than the current findings. On the other hand, our results may not be generalizable to less reliable patients who could not follow the guidance of a very slow dose tapering appropriately.

Third, even though the tempo of dose reduction was carried out at such slow pacing, still, 1 out of 4 patients (6 relapses and 7 resuming baseline doses of 51 GDR patients) failed to reduce any antipsychotic dose. The relapse rate is comparable to that of a recent study on a similar yet smaller population (Huhn et al., 2021). This ratio suggests that discontinuation might not be an ideal goal to pursue for every remitted patient. Indeed, post-reduction CPZE dose > 200 mg/d was identified to be an important factor associated with successful dose reduction (Tani et al., 2020), and the risk of relapse was found to increase disproportionately at the lower doses based on the hyperbolic dose-response curves illustrated by a meta-analysis of antipsychotic dose and relapse prevention (Leucht et al., 2021). As the mean pre-reduction dose of our GDR patients is 200 mg/d, many of them could be regarded as having reached their lowest effective dose before entering this trial at naturalistic setting. Challenging an even lower dose warrants cautious weighing of the risk-to-benefit ratio in this population. Nevertheless, our proposed algorithm provides a practical guide to calibrate the lowest effective dose of individual patients if they wish to taper down further. Additionally, Horowitz et al. (2021a) recently proposed a tapering strategy by cutting off a smaller fraction of antipsychotic recursively at very low dose levels before complete discontinuation of antipsychotics, which might be a solution for these patients.

Fourth, it is encouraging to find that a third of patients could successfully taper antipsychotic doses according to our proposed algorithm to reduce approximately 60% of their baseline doses, suggesting a substantial burden introduced by antipsychotics being lifted. Moreover, patients who could maintain stable under a GDR trial revealed improved clinical severity, personal social performance, and subjective well-being, implying that it is worthwhile to work patiently for desirable outcomes not at the expense of a higher risk of relapse. These patients can still take a comfortable pace in the future, decide if ready to conduct the next dose reduction under supervision, and someday might be able to reach their individual MED, which could be as low as ‘approximate to zero’ [(3/4)*^n^* will be close to 0 when *n* is a big number].

Still, there are several limitations to be addressed. First, our irregular dosing schedule might be inconvenient to some patients, and we have not ascertained medication adherence by testing plasma drug concentrations yet. Second, the difference in relapse rates between groups was small while our sample size was not big enough to support sophisticated analyses. Third, we recognized psychosocial stress as an important risk factor and the soundness of a supportive system as an essential protective factor (Bowtell et al., 2018a), while we did not formally measure these variables. Fourth, most of our patients had more than one psychotic episode, a patient group usually not recommended to discontinue medication by most guidelines (Shimomura et al., 2020). With a history of relapse or long duration of antipsychotic treatment, they might be more vulnerable to relapse during dose reduction and more inclined to retreat to baseline dose once experiencing suspected return of symptoms. Thus, our algorithm might be too conservative for those who only have a single episode or much shorter duration of illness, and our threshold of relapse might be too sensitive to capture those who might be able to regain stabilization without increased dose after one week (Horowitz et al., 2022a).

Echoing Leucht's (2018) opinion, ‘As – due to the subjectivity of psychiatric outcomes – there is room for interpretation, in the future the evidence will have to be presented such that patients can decide (*dose reduction*) themselves.’, our findings suggest how to optimize the risk-to-benefit ratio of using antipsychotics in the protracted course of psychosis. Moreover, our proposed algorithm can instill hope to patients that they can maintain stable remission under a slow and cautious tapering process. Currently, we continue to recruit more eligible patients to undergo this trial and invite those who have completed the first 2-year follow-up to continue the prospective observation. A larger sample size and longer duration of observation can provide more valuable evidence for physicians and patients to create an optimal and personalized approach while considering antipsychotic dose reduction, with a hope of complete discontinuation someday.

## Supporting information

Liu et al. supplementary material Liu et al. supplementary material

## Supplementary material

The supplementary material for this article can be found at https://doi.org/10.1017/S0033291723000429
