# Supplementary material for: Guided antipsychotic reduction to reach minimum effective dose (GARMED) in patients with remitted psychosis: a 2-year randomized controlled trial with a naturalistic cohort
Source: Psychol Med. 2023 Mar 10;53(15):7078–86. doi: 10.1017/S0033291723000429 (PMC10719630; doi:10.1017/S0033291723000429)
Supplement: Liu et al. supplementary material [file S0033291723000429sup001.doc]

Supplement files of a 2-year prospective comparative cohort study (GARMED):

Supplement Figure 1A. Illustration of the first 6 steps of the Cantor ternary set.

[
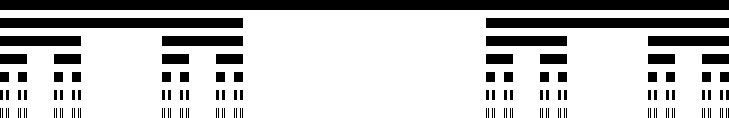
](file://upload.wikimedia.org/wikipedia/commons/5/56/Cantor_set_in_seven_iterations.svg%00塹ᴻ䡿ⲯ嶂藄挧%00%00ꮥ)

Supplement Figure 1B. The evolution of the first 4 steps of the Sierpinski triangle.


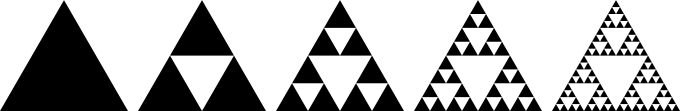


Figure legends:

Supplement Figure 1A: This diagram illustrates how to delete a fraction (for example, one third in the Cantor set) of a line reiteratively. The total length of the remaining segments will reduce gradually and eventually approximate zero, whilst the total number of segments will increase step-by-step as if they are spread everywhere and finally become infinite, an interesting paradox but also a truth.

Supplement Figure 1B: A 2-dimensioned example produced by the same arithmetic rule as the Cantor’s formula is the Serpinski triangle, in which a quarter of the triangle area is erased at a time. Such a re-iterative procedure provides a reasonable template when we try to operationalize a flexible dose reduction algorithm.

Supplement Figure 2. The domain scores checked by PRagmatic-Explanatory Continuum Indicator Summary 2 (PRECIS-2) criteria.


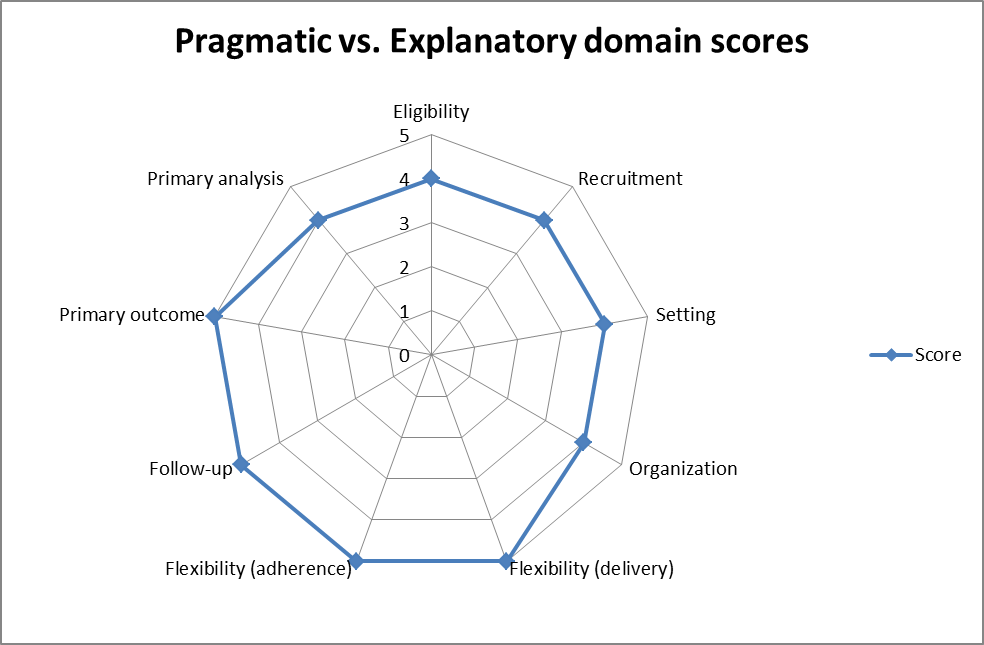


1: Very explanatory; 2: Rather explanatory; 3: Equally pragmatic and explanatory;

4: Rather pragmatic; 5: Very pragmatic

Supplement Figure 3. Illustrations of different trajectories during dose reduction


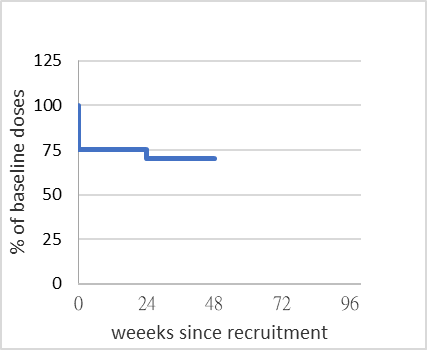


Left Upper: conducting 4 consecutive dose reduction as scheduled

Left Middle: resuming a dose between the lowest point ever reached and baseline

Left Bottom: reducing dose at a tempo slower than the designated schedule

Right Upper: returning to baseline dose for precaution of suspected relapse

Right Middle: relapse, using higher than baseline dose to treat aggravated symptoms

Right Bottom: early exit, no relapse under a dose lower than baseline at the time of losing follow-up

The doses at each timepoint of individual patient were transformed to be the proportions of their baseline doses, that is, the baseline will be 1 uniformly to all patients, whatever doses of antipsychotics received before entering this trial. And then we calculated (current dose)/(baseline dose) to make up a constellation of plots comprised all fractions of doses at each timepoint during the course among all patients with a standardized visual presentation.

Supplement Table 1. Instructions and precautions for conducting guided dose reduction

| Lesson | Content |
| --- | --- |
| Rationale | The rationale for tapering down cautiously to reach a lower effective and acceptable dose and precaution of the risk of relapse |
| Rates of dose reduction | No more than 25% of the current dose will be reduced, as the literature does not support safe reduction by 50% or more at a time |
| Duration of stabilization | No further tapering unless a stable condition has been maintained for at least 6 months after the previous dose reduction |
| The lowest effective dose concept | Not aiming at stopping antipsychotics completely, no matter how low the dose has reached (for example, risperidone 0.5 mg/d or aripiprazole 2.5 mg every second day) |
| Irregular dosing schedule | Keeping a log of the actual dose taken each day as an irregular or intermittent dosing schedule will be used to stay within the proposed dose range |
| Timing of rescue dose | Resuming a rescue dose of antipsychotics (that is, retreat to the previous dose in the algorithm) whenever feel something may be wrong during dose reduction |
| Readiness for next dose tapering | If not feel ready for further tapering for any reason, should stay at the current dose for a longer period before consider the next dose reduction attempt |

Supplement Table 2. Two versions of dosing schedule, one based on a repeating every 4-day cycle (A) and one based on a repeating 4-day alternating with a 7-day cycle (B)

| **(A)** | **Day 1** | **Day 2** | **Day 3** | **Day 4** | **Mean** | **Minimum** |
| --- | --- | --- | --- | --- | --- | --- |
| 6 months | 1 | 1/2 | 1 | 1/2 | 3/4 | 3/4 |
| 1 year | 1 | 1/2 | 1/2 | 1/2 | 5/8 | 9/16 |
| 18 months | 1/2 | 1/2 | 1/2 | 1/2 | 1/2 | 27/64 |
| 2 years | 1/2 | 1/2 | 1/2 | x | 3/8 | 81/256 |

| **(B)** | **D1** | **D2** | **D3** | **D4** | **D5** | **D6** | **D7** | **Mean** | **Minimum** |
| --- | --- | --- | --- | --- | --- | --- | --- | --- | --- |
| 6 months | 1 | 1 | 1 | x |  |  |  | 3/4 | 3/4 |
| 1 year | 1 | 1 | 1 | x | 1 | 1 | x | 5/7* | 9/16 |
| 18 months | 1 | x | 1 | x |  |  |  | 1/2 | 27/64 |
| 2 years | x | 1 | x | 1 | x | 1 | x | 3/7* | 81/256 |

Values provided represent portions of the usually recommended daily dose, while ‘x’ represents no dose given on that day. (A) In practice, it may not be feasible to cut off a quarter piece of a tablet, thus we invented an extended or irregular dosing schedule to meet the dose ranges. (B) If the tablet is indivisible, a drug holiday (also irregular) schedule can be employed to make the average daily dose above the proposed minimum. ‘Mean’ is the average daily dose calculated by having the total doses taken in 4 days divided by four (or total doses taken in 7 days divided by seven). ‘Minimum’ is the lowest dose to reach each step, calculating by the formula (3/4)^n^. The mean must not be below the minimum dose at each step. Besides, long-acting injectable antipsychotics will be given at a longer injection interval as a way to reduce daily dose.

Supplement Table 3. Distribution of baseline antipsychotic use of 96 patients

| Antipsychotics | Number of patients | Dose range |
| --- | --- | --- |
| Amisulpride | 5 | 200-600 mg/d |
| Aripiprazole | 33 | 0.54-20 mg/d |
| Aripiprazole, Long-acting injectable | 1 | 400 mg/q5w |
| Clozapine | 5 | 75-250 mg/d |
| Flupentixol decanoate | 1 | 40 mg/q4w |
| Haloperidol | 2 | 5-15 mg/d |
| Lurasidone | 3 | 60-80 mg/d |
| Olanzapine | 12 | 3.75-10 mg/d |
| Paliperidone | 10 | 3-9 mg/d |
| Paliperidone, Long-acting injectable | 2  1 | 100 mg/q4w  350 mg/q3m |
| Quetiapine | 1 | 25 mg/d |
| Risperidone | 11 | 0.5-6 mg/d |
| Sulpiride | 7 | 50-200 mg/d |
| Trifluoperazine | 1 | 10 mg/d |
| Ziprasidone | 1 | 80 mg/d |
